# Horizontal running inside circular walls of Moon settlements: a comprehensive countermeasure for low-gravity deconditioning?

**DOI:** 10.1098/rsos.231906

**Published:** 2024-05-01

**Authors:** Alberto E. Minetti, Francesco Luciano, Valentina Natalucci, Gaspare Pavei

**Affiliations:** ^1^ Locomotion Physiomechanics Laboratory, Department of Pathophysiology and Transplantation, University of Milan, Milan, Italy

**Keywords:** hypogravity, locomotion, space, bone, artificial gravity, Artemis

## Abstract

Long-lasting exposure to low gravity, such as in lunar settlements planned by the ongoing Artemis Program, elicits muscle hypotrophy, bone demineralization, cardio-respiratory and neuro-control deconditioning, against which optimal countermeasures are still to be designed. Rather than training selected muscle groups only, ‘whole-body’ activities such as locomotion seem better candidates, but at Moon gravity both ‘pendular’ walking and bouncing gaits like running exhibit abnormal dynamics at faster speeds. We theoretically and experimentally show that much greater self-generated artificial gravities can be experienced on the Moon by running horizontally inside a static 4.7 m radius cylinder (motorcyclists’ ‘Wall of Death’ of amusement parks) at speeds preventing downward skidding. To emulate lunar gravity, 83% of body weight was unloaded by pre-tensed (36 m) bungee jumping bands. Participants unprecedentedly maintained horizontal fast running (5.4–6.5 m s^−1^) for a few circular laps, with intense metabolism (estimated as 54–74 mlO_2_ kg^−1^ min^−1^) and peak forces during foot contact, inferred by motion analysis, of 2–3 Earth body weight (corresponding to terrestrial running at 3–4 m s^−1^), high enough to prevent bone calcium resorption. A training regime of a few laps a day promises to be a viable countermeasure for astronauts to quickly combat whole-body deconditioning, for further missions and home return.

## 1. Introduction

The present decade has brought a renewed interest in lunar exploration, with forthcoming missions aimed at returning humans to the Moon’s surface and establishing long-term human settlements [1–3]. Such an ambitious endeavour, however, comes with a multitude of challenges. Among them, the decrease or lack of gravity detrimentally affects astronauts’ cardiorespiratory fitness, musculoskeletal system, and neural control of posture and movement [4–10]. To mitigate these adverse effects and preserve astronauts’ health, exercise-based countermeasures are needed [11–13]. While most of the existing evidence on the field comes from spaceflight and missions on the International Space Station, it is unlikely that exposure to the mild lunar gravity will substantially change these challenges. Indeed, walking in low gravity is hindered by the imbalance between the kinetic and potential energy of the body centre of mass [14–16]. To re-establish that balance, the speed range must be restricted to low values, nullifying its effect for training. Bouncing gaits such as running [17,18], skipping [18–20], and hopping [21] are faster than walking; in lower gravity, however, they may exhibit some mechanical mismatch within the muscle–tendon complex [22,23] and a reduction in their vertical take-off velocity [24]. This can prevent a terrestrial-like, high-impact training stimulus. Additionally, the metabolic demands of bouncing gaits are reduced at Moon gravity [18,21], limiting their potential stimulus for cardiorespiratory fitness.

No consensus has been reached yet about the most effective exercise countermeasures for lunar missions, but studies conducted on Earth and knowledge gained on the International Space Station provide some valuable insights. Low-intensity steady-state exercise or high-intensity interval training on ergometers may serve to preserve cardiorespiratory fitness [12,25–27] but have little impact on muscle and bone mass. Conversely, plyometric exercise on a sledge has shown promising results in preserving cardiorespiratory and musculoskeletal function [28–30] but fails to resemble terrestrial locomotion and may not provide a proper stimulus for keeping balance and motor control. Finally, centrifuge-based methods to emulate increased gravity have some positive impact on muscle function [31]; but Moon-based centrifuges allowing locomotion inside would pose technical challenges and demand substantial electrical energy. Time- and cost-effective ways to emulate terrestrial locomotion on the Moon are hence required.

Here, we propose a novel solution: lunar inhabitants could engage in running on the inside of vertical circular walls, hence running parallel to the Moon surface. Such an activity on Earth is reserved for motorized vehicles during stunt exhibitions (called ‘Wall of Death’ (WoD)) but could be done by humans on the Moon and would generate enough centrifugal acceleration to emulate a higher level of gravity (see §2.5). This would provide an exercise-based countermeasure that simultaneously stimulates cardiorespiratory fitness, the musculoskeletal system and motor control. This article provides a theoretical framework for horizontal circular locomotion, tests its prediction in a terrestrial Moon gravity emulator and estimates its physiological demands.

## 2. Methods

### 2.1. Facility

The ‘WoD’ is an amusement park attraction usually consisting of a ~10 m diameter, ~5 m high cylinder inside which brave pilots horizontally travel on its wall fast enough to prevent downward skidding of their motorbikes. On Earth, this feat is not possible for bipedal muscle-based human locomotion because of the very high running speed needed to ensure a steady performance (see §2.5). In addition, differently from vehicles, in running traction on the ground is discontinuously applied between aerial phases, with a contact time that is shorter at faster running speeds. Lastly, in a WoD, the level path and the inclined ramp to accelerate from zero to the target speed on the wall are quite short.

**Figure 1.**
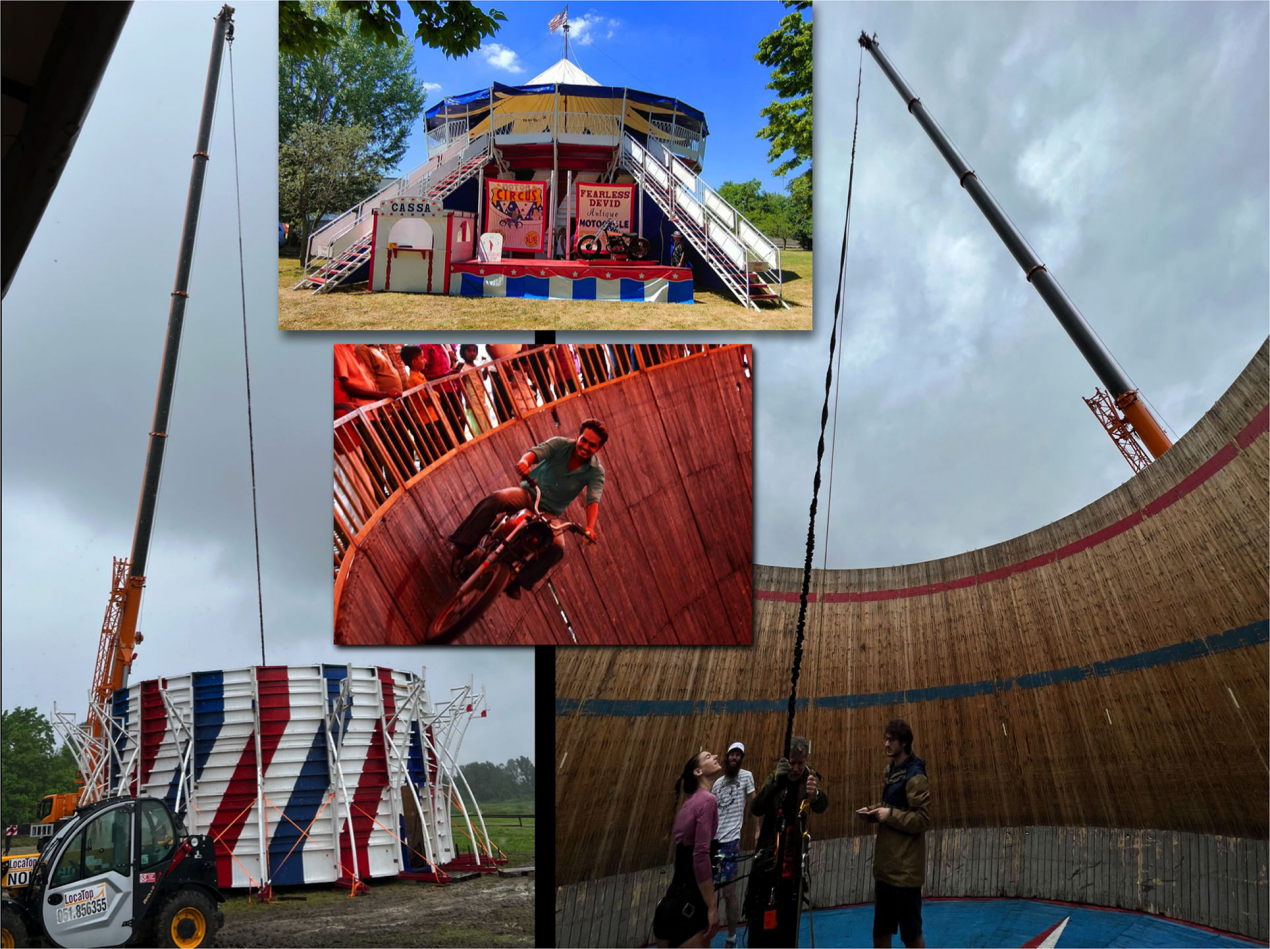
Top inset shows the WoD used in this investigation; left picture represents its ‘naked’ version, with the cap removed as to allow the telescopic crane to hang the body weight unloading bungee cords from a height of 36 m; right picture shows the vertical wall inside the WoD where horizontal running experiments took place at emulated (vertical) lunar gravity; middle inset displays a rider driving fast, with the peculiar upward leaning posture (see §2.5), during a WoD show.

To check whether at a lower gravity, as predicted by physics, humans could confidently run on such a structure, a WoD was hired for one day. The roof of the WoD was removed to allow for a partial weight support system consisting of two in-series bungee jumping rubber bands (Diagoline, Italy, rest length of 4 m). The bungee cord was attached to a 36 m high telescopic crane so as to unload the harness-suspended participants to one-sixth of their weight, to emulate lunar gravity (figure 1).

### 2.2. Participants

Two participants (a 36-year-old man, 1.78 m height, 60 kg body mass and a 33-year-old woman, 1.70 m height, 62 kg body mass) were unloaded as to match Moon body weight using the mentioned bungee jumping rubber bands. Target Moon body weight was one-sixth of that on Earth, checked by a digital scale with the participant lying on the side in the same position as during horizontal running. The harness (Nylon Corset Flying Harness, Climbing Sutra, USA) and the connecting cords to the hip and lower trunk were arranged as to unload the participant from one side of the body both in upright and lateral recumbent positions. In such a horizontal posture, the mobility of the topmost upper limb was limited by the harness cords and its proper swing would have been precluded: hence, that arm did not swing and it handheld the suspension cord.

### 2.3. Data collection

The WoD where participants ran had a radius of 4.73 m and a circumference of 29.7 m; the wall is made of wood, and on the floor, a 0.8 m wide, 30° radially inclined ramp allowed the subjects to accelerate and then run on the vertical wall. Experiments were video-recorded with an iPad Pro (third generation, 11", Apple Inc., USA) and an iPhone 13 at 240 Hz from the centre of the circumference (some recordings were performed also at a normal frame rate, 24 Hz).

On a different day, the same participants ran on a treadmill (PPS 55Ortho, Woodway) at 1*g*, and at 0.8*g*, 0.5*g* and 0.17*g* with a vertical body weight suspension device (LOOP laboratory, ESA Ground-Based Facility for Research in Low Gravity Locomotion, University of Milan) [18,32] at the maximal speed reachable (and held for 10 s) at those gravity levels. Also, the male participant ran (at 1*g*) at 6.69 m s^−1^ along a 40-m hallway equipped with five 3D force platforms (Bertec, USA). The platforms, covering a 4 m long and 0.4 m wide area, detected ground reaction force (GRF) signals to get insight into the mediolateral forces of sequential foot contacts.

### 2.4. Data analysis

In the WoD, tape marks were attached to the wall at a known distance to calculate step length, while movie frame number was used to calculate step frequency and contact/flight time. Running velocity on the (circular) wall was calculated as the product of step length and frequency. Such velocity was used to calculate the artificial, self-generated (horizontal) gravity in the Moon emulated (vertical) gravity environment. Duty factor, namely, the fraction of the stride where the foot is in contact with the ground, allowed estimation of the average vertical (along the craniocaudal body axis) ground reaction force (GRFv) during the contact phase, expressed as a fraction of body weight at the relevant artificial gravity. Peak GRFv values, estimated by modelling the force shape as an almost half-sine wave and considering Nillson & Thorstensson [33] data for the same (terrestrial) running speeds, were 81% higher than average GRFv. Average and peak GRFv values were normalized to Earth body weight (BW_E_) by multiplying them by the artificial gravity and dividing by 9.81 m s^−2^.

### 2.5. Theory

The physics of WoDs is initially quite simple. In the following, we show equations ruling how WoD works when also extra forces, associated with low gravity emulation and body morphology, are involved.

**Figure 2.**
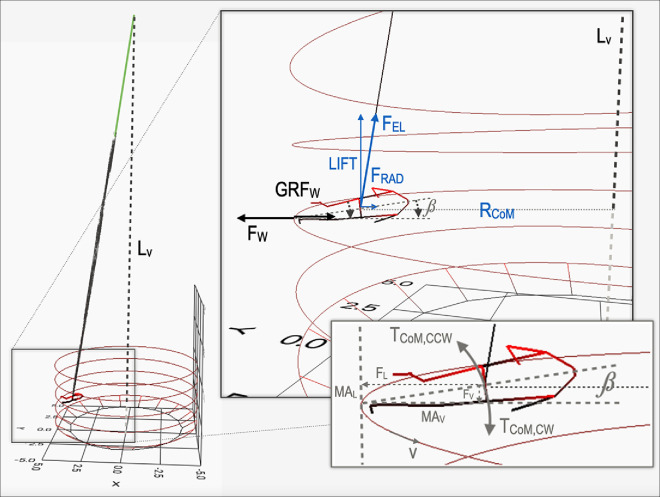
On the left, model simulation (Labview, National Instruments, USA) of running in a lunar WoD. Its enlarged inset, shown on the right-hand side, graphically helps to explain the bias in *v*_min_ introduced by the oblique upward force of rubber bands (see §2.5). Blue graphic elements: *F*_EL_ is the elastic force applied by rubber bands on body CoM, located at a radial distance (*R*_CoM_) from the WoD centre; LIFT and *F*_RAD_ are the orthogonal force components of *F*_EL_ vector, both of them independent of speed. Black graphic elements: *F*_W_ is the centrifugal force transmitted by CoM on the wall, set just by the running speed; GRF_W_ is the ground reaction (centripetal) force, that is, equal to *F*_W_ reduced by *F*_RAD_. In the rightmost inset, the rationale about the body (or rider+motorbike) upwards leaning is illustrated. Grey graphic elements: see §2.5.

**Figure 3.**
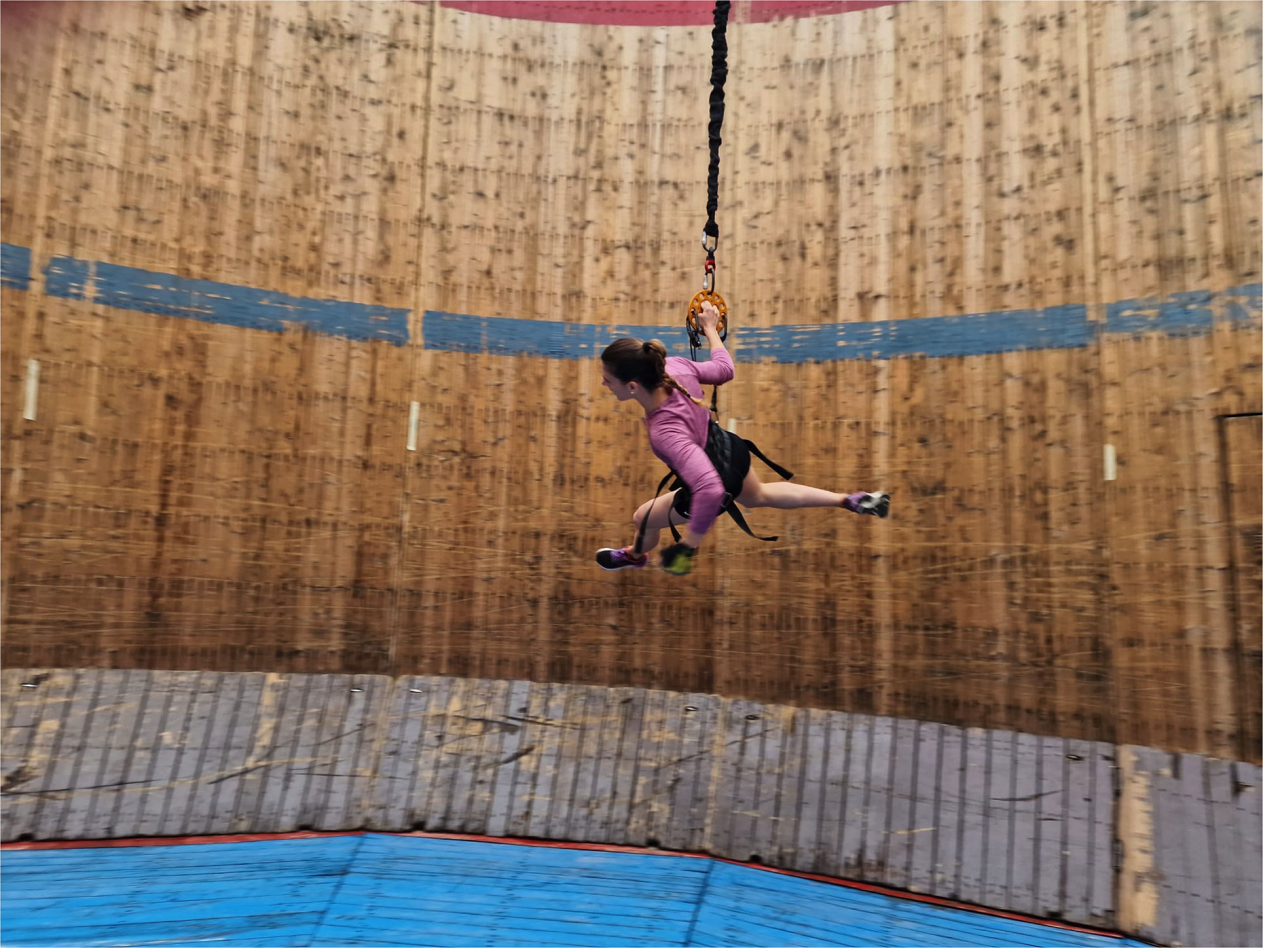
Running horizontally at self-generated artificial gravity in emulated lunar WoD (see also electronic supplementary material, movie).

– A point mass (PM) of mass m horizontally moves at a speed v with a static (vertical) friction coefficient $\mu_{s}$ along a circular path on the internal wall of a cylinder of radius $R_{w}$ without downward skidding if the centrifugal force $F_{c}=\frac{mv^{2}}{R_{w}}$ multiplied by $\mu_{s}$ , that is, the upward resistive force, is equal to or greater than its weight $F_{w}=mg$ , where g is gravity acceleration. This leads to $v_{min,PM}={\left(\frac{R_{w}g}{\mu_{s}}\right)}^{1/2}$ , that is the (mass-independent) minimum speed to ensure a safe WoD performance. On Earth, by using $R_{w}$ =4.7 m and $\mu_{s}$ =0.8 (for rubber wheels against the wooden wall), $v_{min}$ = 7.59 m s^−1^ (27.3 km h^−1^). Both runners and rider+motorbike are far from being a PM, and their overall centre of mass (CoM) is approximately located 0.9 and 0.7 m higher (towards the WoD centre), respectively, than the contact with the ground. The circle travelled by their CoMs, with $R_{CoM}$ =3.8 and 4.0 m, respectively, actually determines $v_{min}$ *on the wall* (those conditions, in the original WoD, correspond to a PM, located in the fulcrum of a massless wheel, where the spokes, rim and pneumatic tyre just transmit the centrifugal force as to cause a centripetal reaction): $v_{min}=\frac{R_{w}}{R_{CoM}}{\left(\frac{R_{CoM}g}{\mu_{s}}\right)}^{1/2}={\left(\frac{R_{w}}{R_{CoM}}\right)}^{1/2}{\left(\frac{R_{w}g}{\mu_{s}}\right)}^{1/2}={\left(\frac{R_{w}}{R_{CoM}}\right)}^{1/2}v_{min,PM}$ resulting equal to 29.6 km h^−1^ for bikers and 30.4 km h^−1^ for runners. The former reports operative speeds ranging from 36 to 50 km h^−1^, with safety factors within 1.22–1.69, the latter realizes that terrestrial running in WoDs is out of question.
– Minimum running speed in lunar WoDs is a different story: one-sixth of *g* makes it theoretically feasible at speeds faster than just 12.5 km h^−1^ (3.5 m s^−1^).
– However, $v_{min}$ can be different from predicted owing to the bias introduced by emulating lunar WoDs on Earth. Pre-tensioned rubber bands, fixed at a height ( $L_{V}$ ) of 36 m above CoM, at horizontal coordinates corresponding to the centre of the WoD, exert a diagonal traction (*F*_EL_) on body CoM (figure 2) so as to generate an almost constant vertical lift equal to five-sixths of terrestrial g . Inevitably, though, a small horizontal force acts towards the WoD centre (*F*_RAD_), decreasing the desired centrifugal effect (*F*_W_) of running on the circular path, and consequently the centripetal ground (wall) reaction force (|GRF_W_|=|*F*_W_|−|*F*_RAD_|). This means that participants’ $v_{min}$ should be faster than predicted: $v_{min}^{corr}=v_{min}{\left(1+\left(1-\frac{g_{M}}{g_{E}}\right)\frac{R_{CoM}}{L_{V}}\right)}^{1/2}$ , where $\frac{g_{M}}{g_{E}}$ is the relative gravity of the Moon, with respect to Earth. The correction factor, according to the present WoD and telescopic crane, is +4.3%, resulting in $v_{min}^{corr}$ =13.1 km h^−1^ (3.63 m s^−1^). It is apparent that, to minimize such a bias, the gravity emulator $L_{V}$ needs to greatly exceed $R_{CoM}$ (×9.5 in the present experiments).
– Another aspect to complete the list of all the force components involved is the upward (rightward) leaning angle (when travelling counter-clockwise, if seen from above) shown by motorcyclists (see lower inset in figure 1) and runners (see the movie in electronic supplementary material). Such a body posture, sketched in figure 2 and shown in figure 3, is necessary to balance the downward/clockwise body torque (*T*_CoM,CW_; on the participant frontal plane), a consequence of the weight of distributed mass along the entire body length, with a slight upward (lateral) tilt ( β ) of the body axis, sufficient to generate an equivalent counter-torque (*T*_CoM,CCW_), namely the product of CoM centrifugal force (*F*_L_, previously called *F*_W_) and the moment arm (MA_L_) issued between its projection on the wall and the point of foot contact. Similarly to the first torque, expressed as $T_{CoM,CW}=F_{V}MA_{V}$ , where $F_{V}=mg_{M}$ and $MA_{V}=\left(R_{w}-R_{CoM}\right)\cos\beta$ , the opposite one is represented by $T_{CoM,CCW}=F_{L}MA_{L}$ , where $F_{L}=\frac{mv^{2}}{R_{W}}$ and $MA_{L}=\left(R_{w}-R_{CoM}\right)\sin\beta$ . By equating the two torques we obtain $\beta =\arctan\frac{R_{W}g_{M}}{v^{2}}$ (exact solution), $\beta =\frac{R_{W}g_{M}}{v^{2}}$ (approximate solution if 0 > β > 30°). To predict the maximum leaning angle, that is, β at $v_{min}$ (as the two variables are inversely proportional), we can use $\beta_{v_{min}}=\arctan\left(\mu_{s}\frac{R_{CoM}}{R_{W}}\right)$ (exact solution), a variable mass and gravity independent, which is equal to the static friction, decreased by a ratio <1 to account for the deviation from the PM model. The previous equations could also be used to infer the speed on the wall from leaning angle data:

$$v={\left(\frac{R_{W}g_{M}}{\beta }\right)}^{1/2}.$$

## 3. Results

Early during the one-day experimental session, participants showed fast familiarization with the unusual horizontal running. This process required only 5–8 attempts and allowed them to start running with no assistance. Participants used the small ramp to accelerate, as the motorbikes do, and ended their performance by safely slowing down their pace and descending from the horizontal posture on the wall down to the upright one on the WoD floor, with no injuries (see figure 3 and electronic supplementary material, movie).

After the familiarization trials, each subject completed seven trials which were then analysed. In each of the trials, participants completed at least an entire circumference/lap (29.7 m), starting after the end of the initial acceleration and ending before the start of the deceleration phase. The ‘grand mean’ (±s.d.) of stride velocity on the wall gave 5.93±0.45 m s^−1^, with a 5.38–6.45 m s^−1^ range; this corresponded to safety factors against the risk of falling within 1.48–1.78, a range similar to Earth WoD motorbikers. The observed ‘grand mean’ speed was achieved with a stride length of 3.78±0.29 m, a stride frequency of 1.58±0.17 Hz, a contact time of 0.176±0.017 s and a duty factor of 0.27±0.01.

**Figure 4.**
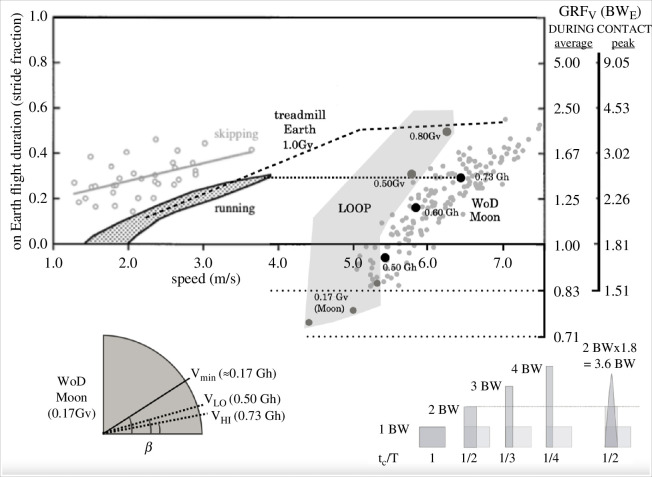
Main upper graph. The graph is combined with a previously published one [19], relating total flight duration ( $t_{f}$ , as a fraction of the stride cycle T ), in terrestrial running and skipping at different speeds, to the consequent average (along the craniocaudal body axis) $GRF_{V}$ ( $\bar{GRF_{V}}$ , expressed in BW_E_) during the contact phase (though equation $\bar{GRF_{V}}=\frac{1}{1-\frac{t_{f}}{T}}=\frac{1}{2\;df}$ , where df is the duty factor). With average $GRF_{V}$ , the second vertical axis on the right-hand side of the graph is the peak $GRF_{V}$ during contact, $\hat{GRF_{V}}=1.81\;\bar{GRF_{V}}$ , where the constant has been reported in detailed investigations about terrestrial running, also at high speeds [33]. Such a process is graphically illustrated in the sketches at the bottom-right zone of the figure, where $\bar{GRF_{V}}$ during contact phases increases when its duration decreases (the last sketch illustrates the effects of considering peak rather than average force). With respect to the original graph, data from the present experiments have been added for running speeds in the range 4.4−7.5 m s^−1^, where $GRF_{V}$ values, in order to be graphed as BW_E_ fractions, have been multiplied by BW_A_/BW_E_, where BW_A_ is body weight at the speed-driven artificial gravity. Big grey circles (enclosed in the light grey area) refer to treadmill data in the LOOP laboratory facility, where gravity was vertically emulated at lunar level (the three lowest points) and at 0.50*g* and 0.80*g* levels (vertical gravity, hence the ‘v’ subscript). Large black and small grey circles refer to actual WoD experiments: the former represents the average $GRF_{V}$ data calculated in three distinct trials, namely, at slow, medium and high speeds (where the artificial (horizontal, hence the ‘h’ subscript) gravity resulted to be 0.50*g*, 0.60*g* and 0.73*g*), while the latter shows individual stride values. Finally, the bottom-left zone of the figure graphically shows the expected leaning angles (from the horizontal) of the body axis in the three relevant conditions of emulated lunar WoD: minimum, low and fast running speeds.

**Figure 5.**
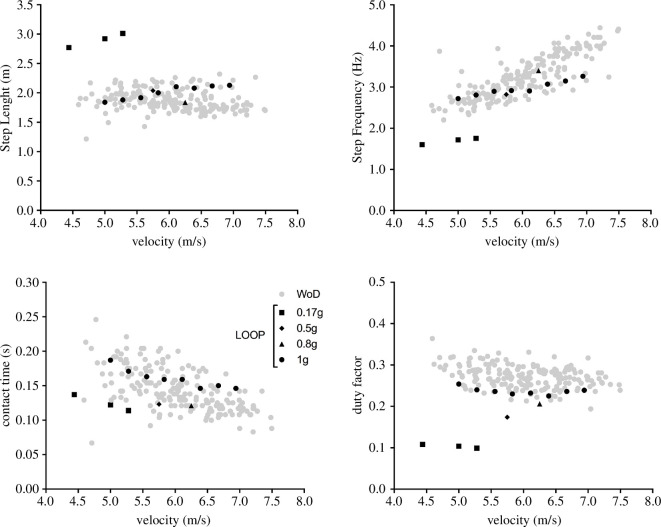
Kinematics of running as a function of speed at different gravity levels. Black-filled symbols refer to treadmill (upright) running: circles—Earth gravity; squares, diamonds and triangles—Moon, 0.5*g* and 0.8*g* in the LOOP laboratory. Grey circles: horizontal running in the (vertically) emulated lunar gravity WoD.

These velocities produced a (predicted) leaning angle of 12.4°±1.9° (range 10.4°–14.9°) with a stance-average vertical (along the craniocaudal body axis) GRF (in terms of BW_E_; see figure 4) of 1.13±0.16 (range 0.93–1.34) and a peak of 2.04±0.29 (range 1.67–2.42). A total of 194 steps were analysed and, when pooled together, covered an even wider range of velocity (4.60–7.50 m s^−1^) (figure 5). Like typical Earth running, faster running velocities were achieved primarily via higher step frequencies (step length was quite constant), while contact time and duty factor decreased with velocity (figure 5).

In the LOOP laboratory session, the maximal running speed achievable on the treadmill by participants was 5.28 m s^−1^ at Moon gravity, 5.75, 6.25 and 6.94 m s^−1^ at 0.5*g*, 0.8*g* and 1*g*, respectively; the associated values of stride length, frequency, contact time and duty factor are reported in figure 5. Measured average and peak vertical ground reaction forces (in terms of BW_E_) at the maximal speed were 0.85 and 1.54 at 0.17*g*, 1.44 and 2.60 at 0.5*g*, 1.94 and 3.51 at 0.8*g*, 2.09 and 3.79 at 1*g* (figure 4). Kinematics of running at (vertically) emulated Moon gravity in LOOP was markedly different from those during (horizontal) self-generated gravity in the WoD and the other emulated gravity levels. Step lengths were substantially longer, and the duty factor was greatly reduced on the actual Moon (figure 5), whereas, as partially expected, running kinematics at 0.5*g* and 0.8*g* were closer to 1*g* and WoD steps data (figure 5).

## 4. Discussion

We have demonstrated for the first time that humans can safely run horizontally in low gravity conditions inside a cylinder, sized as a terrestrial ‘WoD’, through a speed-driven, self-generated higher artificial gravity. The following discussion focuses on whether this opens a new multi-system countermeasure against deconditioning during long-term exposure to low gravity.

It is important to note that the gait patterns of upright running on Earth and horizontal low gravity running in the WoD are quite similar, as witnessed by the kinematics results. With the observed leaning angle ( β ), the WoD running pattern would correspond on Earth (see electronic supplementary material) either (i) to a partially unloaded treadmill running (with more bent knees) affected by a constant side wind at a speed only proportional (through body lateral projection area) to emulated low gravity and the participant’s mass, or (ii) to a circular running [34] on a ground path with radius in the range 4.6–14.2 m. Those analogies in the leaning angles are meant to suggest potential training schemes on Earth when emulated low-gravity WoDs are not at hand.

To be a compound and comprehensive countermeasure, the proposed activity has to aim at the most relevant deconditioning targets, namely the musculo-skeletal-neural and cardio-respiratory districts.

### 4.1. Muscle

Because of the flight phases and the horizontal posture, WoD running occurred at a much faster speed than *v*_min_, that is the minimum speed to avoid downward skidding (see safety factor values in §2.5). Running speeds ranging from 5 to 7 m s^−1^, with the associated high step frequency (2.5–4 Hz), are likely to require a high mechanical external work from the muscle–tendon unit, which is only partially mitigated by artificial (lateral) gravities that are lower than on Earth. The work required to move the limbs relative to the overall body CoM, the so-called kinetic internal work (*W*_INT,K_) [35], and to restore kinetic energy loss owing to the damping effect of joint friction (*W*_INT,F_) [36], could be even higher as well, depending on stride frequency and speed but not on (the lower than Earth) gravity. Thus, the total mechanical work that the muscle–tendon units have to perform is supposed to be higher than the work that the same actuators would generate when running (upright) on the lunar surface [18].

Another effect to be considered is the extra mediolateral GRF (GRF_L_, vertical with respect to WoD) each foot must exert during the contact phase, with respect to emulated Moon gravity, as caused by the short duty factor (= 0.27 at 6 m s^−1^) of fast running. By following the same rationale as discussed in the caption of figure 4:


$$\bar{\frac{{GRF}_{L}}{{BW}_{M}}}=\frac{1}{2\;df}\;and\;\frac{BW_{M}}{BW_{E}}=\frac{1}{6}\;,thus\;\bar{\frac{GRF_{L}}{BW_{E}}}=0.31.$$

With our participants’ mass, $\bar{GRF_{L}}$ would correspond to about 186 N, which is not a negligible force when considering that in terrestrial running at the same speed its value is about 102 N. Also, the (lateral) Moon gravity in the WoD asymmetrically affects the mediolateral forces of the feet that, in the absence of it, would show the same average net ‘push’ toward the body CoM. Rather, based on our experiment on the ground-mounted force platforms, WoD counter-clockwise running is expected to increase the average left foot push to about 256 N and decrease the right one to about 54 N. Such a greater effort, together with the introduced foot force asymmetry, points out the higher force demand out of leg propulsive muscles and the need to train in WoDs in both clockwise and counter-clockwise directions. As discussed in the electronic supplementary material, this extra force caused by the leaning angle could be achieved by astronauts on Earth when low gravity WoDs are not at reach, by running along circles or running straight on a treadmill with lateral wind.

**Figure 6.**
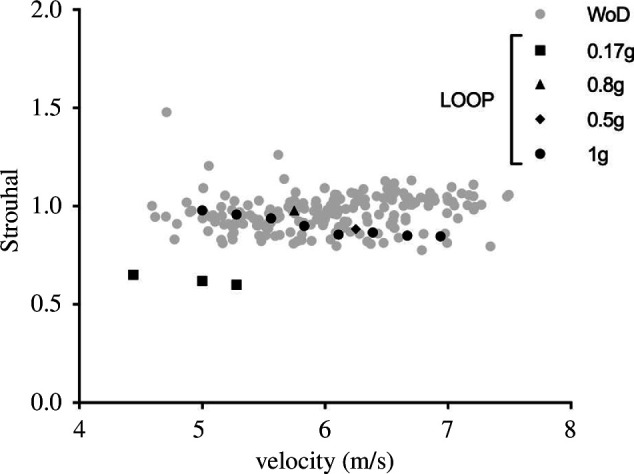
Strouhal number, an index of dynamic similarity for bouncing gaits, as a function of speed at different gravity levels. Symbols as in figure 5.

In order to indirectly check whether bouncing gaits like running in both WoD (horizontally) and LOOP (upright) are dynamically similar [37], Strouhal dimensionless index has been computed as $Str=\frac{f\times l}{v}$ , where f is stride frequency, l is leg length and v is running speed. By quoting Alexander’s work, since v/f corresponds to stride length, a constant *Str* means a given constant ratio between leg length and stride length, regardless of speed. The results confirm that lunar WoD running at self-generated artificial gravities is dynamically similar to usual terrestrial running, with *Str* ≈ 0.80–1.10 (see figure 6). The same range was obtained in LOOP for gravities ≥0.50*g* only, emphasizing the unorthodox dynamics of (upright) running at high speeds and Moon gravity (*Str* ≈ 0.55–0.65).

A similar hyper-gravity approach was proposed by Picerno [38,39] to train track and field sprinters on Earth by having them run on an inclined circular ramp (from 34° to 60°, ‘centrifugal track’). No attempts were performed at 90°, as rightly assumed unfeasible for human running. It seems surprisingly convenient that an unfeasible locomotion on Earth can generate higher artificial gravity, thus a higher training stimulus, on the Moon.

### 4.2. Bone

In the randomized controlled trial scheme by Kramer and colleagues [29] in healthy participants undergoing 60 days of bed rest, 48 sessions of jump training (or an average of 0.8 sessions per day) prevented many aspects of body deconditioning, including the loss in bone mineral density and calcium content. On average, the sessions consisted of 78 jumps each, with a GRF peak of 3.6 kN (mean among participants) corresponding to 4.8 BW, or 2.4 BW for each limb. Assuming running at 6 m s^−1^ in the WoD with a peak GRF per step of 2.2 BW (see figure 4), the number of steps required to obtain an equal cumulative GRF for a session is (78 × 2 × 2.4/2.2 =) 170. With a WoD circumference of 30 m and a step frequency of 3.2 Hz (see figure 5), one lap at that speed lasts 5 s and is composed of 16 steps. Hence, a total of 10–11 laps every 1.25 day (the time equivalent of one session), or 8–9 laps every day, for a total exercise duration of 40–45 s, allows getting a summed GRF of the same extent as that of Kramer *et al*. [29]. This would grant lunar WoD runners the same osteogenic effect observed in that study. Furthermore, previous evidence-based training rules [40,41], from animal and human models, assuring to elicit an effective bone strength maintenance have here been checked: (i) high impact dynamic loading, rather than static, (ii) no more than 40–60 ground contact phases per day (in WoD running, every single leg impacts at ‘stride frequency’, for a total of 64–72 loading cycles = 8–9 laps times 8 strides per lap), and (iii) avoiding prolonged training sessions that could make bone cells accommodate to a given loading regime, resulting less responsive to further loading signals (here 40–45 s per day, which could be partitioned, also owing to the high metabolic intensity—see §4.4—into no more than two sessions [42]). Also in this case, horizontally running in a lunar WoD seems to offer a higher ‘re-conditioning’ potential, when compared with upright running or hopping on the lunar surface, in promoting effective bone remodelling via greater ground reaction forces as owing to the self-generated artificial gravities.

### 4.3. Neural

As mentioned, the running pattern in a hypogravity WoD is similar to the typical running pattern on Earth, as shown by the kinematics results and a dynamic similarity index. A high number of joints is involved, thus a whole-body activation of locomotory/balance actuators is issued. During unconstrained motion on the Moon (in our emulated lunar WoD, the only constraints were the harness and an upper limb hanging from the bungee bands) neural integration takes care of both propulsion and posture maintenance within narrow operative ranges [43]. Differently, for example, plyometric sledge ergometers [28] reduce the degrees of freedom of the body by constraining the trunk to rest on the sledge during the repeated jumps, with no involvement of the many postural muscles of the trunk and the dynamic balance of a gait task. Similar comments apply to cycloergometers [25].

### 4.4. Cardio-respiratory

The specific literature about exercise countermeasures strategy to hypogravity deconditioning in terms of cardio-respiratory/metabolic health is controversial [44] and the effects of exercise type (i.e. aerobic or resistance training) and of its dose (i.e. intensity, duration and frequency) are still debated [45]. Papers about high intensity interval training (HIIT) seem to indicate that the metabolic power of the bouts, to elicit an improvement in cardio-circulatory fitness, should be higher than 90% of $\overset{˙}{V}O_{2max}$ [46]. Although oxygen consumption was not measured in the present study, it is possible to estimate the (minimum) metabolic effort of WoD running at the self-generated artificial gravities by considering the following rationale:

(i) The metabolic cost of terrestrial running is remarkably constant (*C* ≈ 4.0 J kg^−1^ m^−1^) for speeds ranging from 2.5 to 7.3 m s^−1^ (this comes from treadmill studies and from the world records in a distance range from 5000 m to the marathon [47]). (ii) We know, by using the regression equation of metabolic data of running at different emulated gravities (Earth, Mars and Moon), that *C* values show the same speed-independence in the range 1.5–3.5 m s^−1^ [18] at each gravity level, with a trend of each average *C* value to linearly decrease with gravity. (iii) In the absence of experimental metabolic data at speeds higher than the investigated range, a first hypothesis is that running *C* when on the Moon and Mars will be at least as high as at lower speeds, as occurring in the extended and investigated speed range on Earth. Such a hypothesized minimum *C* is certainly to be exceeded in WoD running for the many reasons already exposed in the present study, among which is the needed extra force from propulsive muscles. (iv) By interpolating *C* values from the equation published in Pavei & Minetti [21] for the artificial gravity values corresponding to the running speed range adopted in WoD (0.5*g*–0.73*g*), and multiplying each of them by the relevant speed (5.5 and 6.5 m s^−1^, respectively), the corresponding ${\dot{V}O}_{2}$ can be obtained; the final values, including rest metabolism, sum up to 49 and 67 mlO_2_ kg^−1^ min^−1^. (v) Artemis Program crew members are expected to be trained on Earth as to reach about 40 mlO_2_ kg^−1^ min^−1^ of $\overset{˙}{V}O_{2max}$ pre-flight, as reported for International Space Station astronauts [9]. Thus, WoD ${\dot{V}O}_{2}$ would correspond to 123–168% of $\overset{˙}{V}O_{2max}$ ; with these intensities and the training duration reported above for the bone remodeling, WoD horizontal running could be addressed as a particular regime of HIIT [46]. It is worth recalling that, as anticipated, these are probably underestimated values needing further experiments in WoD to be properly assessed.

Countermeasures for the listed physiological districts, devoted to fight low gravity-specific impairments, are not always supposed to overlap in terms of training modality and regime. We suggested that the daily sets of running bouts in a lunar WoD fulfilling bone strength needs are also compatible with cardiovascular fitness maintenance. Although other investigations, such as bed-rest studies in the proximity of a Moon emulated WoD, will deepen and refine our search for the most comprehensive and feasible countermeasure there, we can be confident that also many body muscles, other than just propulsive ones, and their neural activation could be preserved by the gait proposed in the present study.

When on the Moon, as previously discussed, the exposition to real gravity will benefit from removing emulation biases: (i) higher tangential speed owing to the inevitable upward (centripetal) component of body unloading rubber traction force; (ii) limbs experiencing residual Earth gravity, particularly during swing; and (iii) the extra body mass owing to harness, cords and suspension gear (almost 9 kg) to be accelerated along the circular path, an effect here not considered. In addition, a much greater familiarity induced by a purposely designed pre-flight training of horizontal locomotion will contribute, in a (likely smaller) WoD on the Moon, to move at slower speeds.

Finally, by realizing the logistic restrictions in bringing/building dedicated gym machines/facilities on the Moon, it is worth mentioning that the described countermeasure could be assured by running horizontally on the circular wall of astronauts’ inhabited settlements, with no need for extra electrical supply.

In conclusion, while being aware of the small sample size, of the crudeness of kinematics acquisition in such a peculiar field experiment, and that dedicated bed rest studies will be needed to refine this topic, we are confident in our findings: (i) on the Moon, where normal (vertical) running is impaired owing to low gravity, humans can steadily use this gait horizontally in a WoD, an impossible feat on Earth, and (ii) the reached speeds create a sufficiently high (lateral) self-generated artificial gravity likely capable of maintaining, through a few short, almost ‘terrestrial’ running laps a day, an acceptable cardio-motor fitness and bone mineral status, useful to locally move and work around, to prepare the long trip to Mars, and to return home in good condition. All of this, by using an inexpensive and passive facility already built in their circular inhabited units.

## Data Availability

Data are available as electronic supplementary material [48].
